# Discrimination between Newly Formed and Aged Thrombi Using Empirical Mode Decomposition of Ultrasound B-Scan Image

**DOI:** 10.1155/2015/403293

**Published:** 2015-01-28

**Authors:** Jui Fang, Yung-Liang Wan, Chin-Kuo Chen, Po-Hsiang Tsui

**Affiliations:** ^1^Ph.D. Program in Biomedical Engineering, College of Engineering, Chang Gung University, Taoyuan 33302, Taiwan; ^2^Department of Medical Imaging and Intervention, Chang Gung Memorial Hospital at Linkou, Taoyuan 33302, Taiwan; ^3^Department of Medical Imaging and Radiological Sciences, College of Medicine, Chang Gung University, Taoyuan 33302, Taiwan; ^4^Medical Imaging Research Center, Institute for Radiological Research, Chang Gung University and Chang Gung Memorial Hospital, Taoyuan 33302, Taiwan; ^5^Department of Otolaryngology, Head & Neck Surgery, Chang Gung Memorial Hospital and Chang Gung University, Taoyuan 33302, Taiwan

## Abstract

Ultrasound imaging is a first-line diagnostic method for screening the thrombus. During thrombus aging, the proportion of red blood cells (RBCs) in the thrombus decreases and therefore the signal intensity of B-scan can be used to detect the thrombus age. To avoid the effect of system gain on the measurements, this study proposed using the empirical mode decomposition (EMD) of ultrasound image as a strategy to classify newly formed and aged thrombi. Porcine blood samples were used for the *in vitro* induction of fresh and aged thrombi (at hematocrits of 40%). Each thrombus was imaged using an ultrasound scanner at different gains (15, 20, and 30 dB). Then, EMD of ultrasound signals was performed to obtain the first and second intrinsic mode functions (IMFs), which were further used to calculate the IMF-based echogenicity ratio (IER). The results showed that the performance of using signal amplitude of B-scan to reflect the thrombus age depends on gain. However, the IER is less affected by the gain in discriminating between fresh and aged thrombi. In the future, ultrasound B-scan combined with the EMD may be used to identify the thrombus age for the establishment of thrombolytic treatment planning.

## 1. Introduction

Ischemic stroke, deep vein thrombosis (DVT), and pulmonary emboli (PE) are deadly diseases caused by thrombi. For example, DVT annually occurs in approximately 2 million Americans [1]. More than 10000 people die from PE in the United States every year [2]. For these reasons, noninvasive early detection of thrombus is of great importance in clinical diagnoses. Various clinical imaging methods can be applied to screen and detect thrombi; however, once thrombi are detected, aged thrombi must be differentiated from newly formed thrombi. This is because clinical therapies and thrombolytic agents' responses to thrombolysis depend on the thrombus age [3, 4]. Aged thrombi are more resistant to thrombolysis than fresh thrombi [5–8]. Thrombi appearance may predict the efficacy of thrombolysis [9]. In particular, evaluating the thrombus age may help assess treatment efficacy and permit the customization of therapeutic procedures.

Among all the imaging modalities, ultrasonic imaging is more convenient and suitable for routine clinical evaluation of thrombi age because of its low cost, short examination time, and real-time capability. Ultrasonography (B-mode image), which combines duplex ultrasound imaging with compression technique, is clinically employed to examine the existences of thrombi [10, 11]. In fact, ultrasound imaging is also possible to have the ability to discriminate between fresh and aged thrombi. During thrombus aging, the proportion of red blood cells (RBCs) in the thrombus decreases. This is because of thrombi retraction, which squeezes RBCs and other contents out of thrombi [12, 13] so that ultimately only fibrin remains [14, 15]. It can be expected that the backscattered ultrasound signals from RBCs and the corresponding signal intensity may be affected by the change in the number of RBCs in the thrombus during aging. In other words, analyzing the signal amplitude (or image intensity) of ultrasound B-scan image may be the most convenient method for evaluating the structure and age of the thrombus.

An annoying problem may be encountered when using the B-scan to identify the thrombus age. The image intensity is determined according to the amplitude of the backscattered signals resulting from the interactions between acoustic scatterers and the incident wave. In addition to the effects of an acoustic impedance mismatch, the brightness of B-scans depends on system factors such as the system gain, time gain compensation (TGC), log compression, and signal/image processing. Among these, the system gain is the dominant factor for determining the amplitudes of signals and the corresponding image brightness. The most frequent problem in clinical settings entails different users using different gains for screening, thus, resulting in varied explanations for the echogenicity of examined tissues. For a robust assessment of thrombus age by the conventional ultrasound B-scan, a method that can describe the signal intensity without a significant effect of system gain on the measurement is highly required.

Recently, the Hilbert-Huang transform (HHT) has become a highly attractive time-frequency analysis method for nonlinear and nonstationary data [16, 17]. In the HHT, the empirical mode decomposition (EMD) is developed to decompose a signal into a set of intrinsic mode functions (IMFs) for calculating the instantaneous frequencies through the Hilbert transform. The advantage of EMD over the other signal decomposition methods is that EMD requires no signal base for decomposition; therefore, a signal can be adaptively decomposed into many signal components. Also, the IMFs obtained from EMD of a signal come from the same measurement system, and thus they will have the same gain effect. Using the IMFs for normalization may allow the evaluation of tissue echogenicity with a reduced gain effect.

The objectives of this study is (i) exploring the feasibility of using the intensity of ultrasound B-mode image to discriminate between newly formed and aged thrombi and (ii) proposing a new index based on the EMD for describing the echogenicity of thrombus with a reduced effect of system gain. In the next sections, we will briefly review the theoretical background of EMD and explain the details for sample preparations, experimental procedures, and data analysis. The experimental results are used to discuss the potentials of the proposed method in future clinical applications.

## 2. Theoretical Background

EMD is a key constituent of the HHT, an adaptive time-frequency analysis method for nonlinear and nonstationary data [16, 17]. We briefly explain how to perform the EMD as follows.

First, we determine the local maxima and minima of a signal *x*(*t*) and use cubic spline interpolation to obtain its upper and lower envelopes. If the mean of these two envelopes is *d*_1_(*t*), then the difference between the signal and *d*_1_(*t*) is the first component *h*_1_(*t*) as follows:

(1) $$\begin{array}{c} h_{1}\left(t\right)=x\left(t\right)-d_{1}\left(t\right). \end{array}$$

This is called the sifting process. To determine whether *h*_1_(*t*) is an IMF, it must be a single-component signal that fulfills the following conditions: (i) the number of zero and extreme crossings must be no more than one in the entire data set and (ii) the mean value of the upper and lower envelopes, which are defined using the local maxima and minima, respectively, is zero at any time, meaning that the two envelope curves are symmetrically about the time axis. Ideally, when the cubic spline interpolation is perfect, with no gentle hump on the signal slope, *h*_1_(*t*) must satisfy the IMF requirements. However, imperfect fitting commonly produces overshoots and undershoots that generate new extrema and shift or exaggerate existing ones. Even when the fitting is perfect, humps may become local extrema after the first round of sifting. Moreover, the envelope mean may differ from the true local mean of the signal for nonstationary data, resulting in an asymmetric waveform. Therefore, the sifting process must be repeated *k* times until the extracted difference is an IMF. In the second iteration, *h*_1_(*t*) is treated as the original data in the second sifting process:

(2) $$\begin{array}{c} h_{1}\left(t\right)-d_{11}\left(t\right)=h_{11}\left(t\right). \end{array}$$

The sifting process is repeated *k* times until *h*_1*k*_(*t*) is determined, which is an IMF:

(3) $$\begin{array}{c} h_{1\left(k-1\right)}\left(t\right)-d_{1k}\left(t\right)=h_{1k}\left(t\right). \end{array}$$

Then, we define

(4) $$\begin{array}{c} c_{1}\left(t\right)=h_{1k}\left(t\right) \end{array}$$

as the first IMF component (i.e., component C1) for the data. Overall, C1 contains the finest and shortest period component of the signal. Subsequently, we can subtract *c*_1_(*t*) from the signal:

(5) $$\begin{array}{c} x\left(t\right)-c_{1}\left(t\right)=r_{1}\left(t\right). \end{array}$$

Because residue *r*_1_(*t*) still contains information about the components with longer periods, we consider it new original data and apply the same aforementioned sifting process. This procedure can be repeated for all subsequent *r*_*j*_(*t*) values, yielding

(6) $$\begin{array}{c} r_{1}\left(t\right)-c_{2}\left(t\right)=r_{2}\left(t\right),\ldots ,r_{n-1}\left(t\right)-c_{n}\left(t\right)=r_{n}\left(t\right). \end{array}$$

Summing (4)–(6) ultimately yields

(7) $$\begin{array}{c} x\left(t\right)=\overset{n}{\underset{i=1}{\sum }}c_{i}\left(t\right)+r_{n}\left(t\right). \end{array}$$

This indicates that *x*(*t*) is decomposed by EMD into *n* IMFs (C1, C2, C3,…, C*i*, from high-frequency to low-frequency components) and a residue *r*_*n*_(*t*), which is the signal trend with a maximum of one extremum or a constant.

## 3. Materials and Methods

### 3.1. Preparations of Fresh and Aged Thrombi

Fresh porcine blood containing 15% acid citrate dextrose anticoagulation solution was collected from a local slaughterhouse. The blood was passed through a sponge to filter out impurities and was discarded whenever coagulation was observed. The blood was centrifuged at room temperature for 15 min at 1500 rpm to separate the RBCs from the plasma. Whole blood was prepared by adding RBCs to plasma for hematocrits of 40%. The blood was then added to a container, which was placed on a stirrer to be stirred for 15 min and bathed in 37.3°C water for 20 min while exposed to room air to equilibrate the gas content. Fresh thrombi were initiated by the addition of 0.5 M CaCl_2_ to a ratio of 1 : 10 [18, 19] and molded in a 6 mL syringe, which was incubated at 37°C for 3 hours during coagulation. Then, thrombi samples were placed in a 37°C saline bath for 5 days to induce spontaneous retraction. The above model for thrombus aging is based on the single tube method, which is typically a clinical retraction test method [20]. Fresh and aged thrombi were made from six animals, respectively (*n* = 12).

### 3.2. Histological Observations

To confirm the change in the number of RBCs with increasing the thrombus age, each thrombus was processed using hematoxylin and eosin stain (H&E stain). At first, each thrombus was immersed in 10% formaldehyde buffered solution for 24 hours. Thrombi were then paraffin-embedded, cut into 5 *μ*m sections, and stained with H&E. Histological images of thrombi were photographed using a microscope with a color digital camera (400x magnification). To further confirm fibrin deposition in the thrombus after thrombus aging, the thrombi samples were also sent to the Department of Pathology in Chang Gung Memorial Hospital for taking images using a scanning electron microscopy (SEM; Model S-3000N, Hitachi, Tokyo, Japan). An experienced pathologist was invited to examine both the H&E stained and SEM images to confirm the changes in the structures of fresh and aged thrombi.

### 3.3. Data Acquisition and Analysis

**Figure 1:**
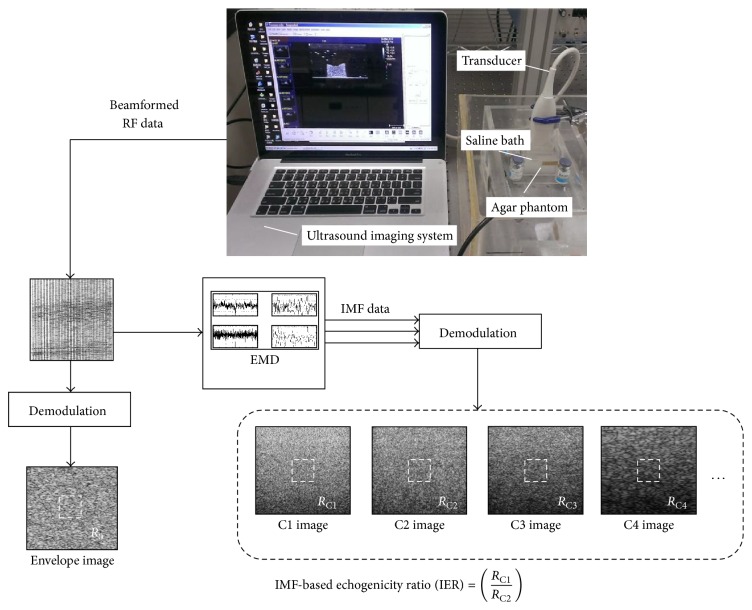
Illustration to explain the IER calculation. The raw RF image data were decomposed into the IMFs by using the 2D EMD. IER was defined using the first (C1) and second (C2) IMFs for signal normalization.

Figure 1 illustrates how we performed ultrasound measurements of thrombus and acquired image data for analysis. Each thrombus sample was placed on the agar phantom in an acrylic case filled with saline solution. The agar phantom was made by boiling the agar-water mixture (dissolving 0.75 g of agar powder per 100 mL of water) and cooling it to form a solid gel. An agar phantom was used as a bottom layer to prevent an overlap of strong reflection signals from the bottom of the case with backscattered signals from the thrombus. Each thrombus was scanned using a commercial system (Model 3000, Terason, Burlington, MA, USA) equipped with a 7.5 MHz linear array transducer (Model 10L5, Terason, Burlington, MA, USA) to acquire raw beamformed radiofrequency (RF) data consisting of 256 scan lines of backscattered signals. Totally three system gains were used to collect data, including 15, 20, and 30 dB. The focal zone was adjusted to locate at the central part of the thrombus sample to reduce the effect of beam diffraction. The time gain compensation (TGC) was not used. The sampling rate of RF signals was 30 MHz. A total of five independent scans were performed on each thrombus.

For each image RF data, scan lines were demodulated using the Hilbert transform to construct the envelope image, and the B-mode image was formed using a logarithm-compressed envelope image (dynamic range = 40 dB). In the meantime, the raw RF data were decomposed into the IMFs by using a two-dimensional (2D) EMD, which was developed by Wu et al. [21] for image EMD processing. The 2D EMD programs used in the study were obtained from the website of the Research Center for Adaptive Data Analysis, National Central University, Chungli, Taiwan (http://www.ncu.edu.tw/~ncu34951/). After applying 2D EMD on the RF signals, each IMF signal was demodulated and log-compressed to display the IMF-based B-mode images (i.e., C1, C2,…, C*i* images). Refer to Figure 1. A 1 × 1 cm sized region of interest (ROI) was applied to calculate the average envelope amplitude of the B-mode, C1, and C2 images. Then, an IMF-based echogenicity ratio (IER) was defined by

(8) $$\begin{array}{c} \text{IER}=\frac{R_{\text{C}1}}{R_{\text{C}2}}, \end{array}$$

where *R*_C1_ and *R*_C2_ represent the average envelope amplitude of the C1 and C2 images, respectively. Actually, the IER is a C1-C2 normalized intensity ratio. The reason why we selected the C1 and C2 images is that higher-frequency IMFs have smaller signal attenuations, larger signal amplitudes, and relevant physical meanings [22]. EMD produces several subsignals with the same gain effect, and the subsignals can be used for normalization to eliminate the gain effect.

The envelope amplitude values of the B-scan and the IER obtained under different system gains were used to evaluate (i) the feasibility of using B-mode image to discriminate between fresh and aged thrombi and (ii) the performance of using the proposed IER to reduce the gain-dependence for reflecting thrombus aging. Data were expressed by mean ± standard deviation. Independent *t*-test was used to calculate the probability value (*P* value) for evaluating the statistical significance.

## 4. Results

**Figure 2:**
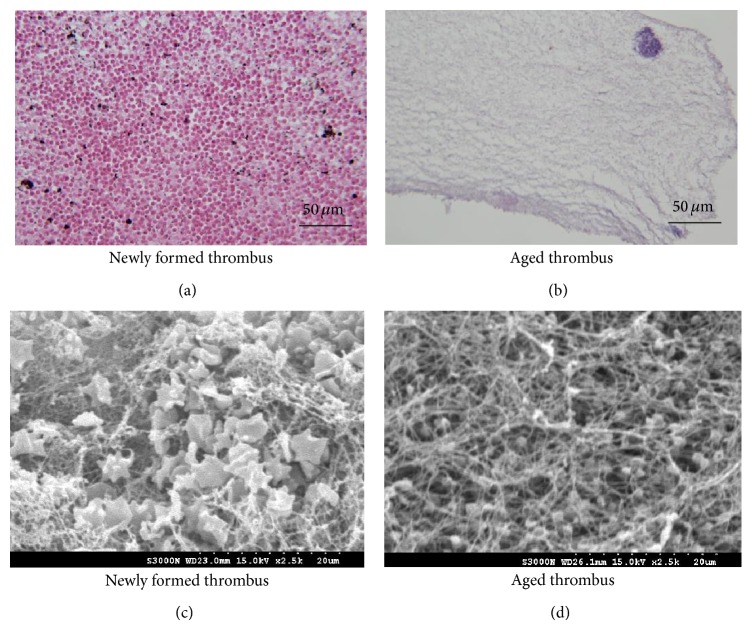
(a) and (b) H&E staining of fresh and aged thrombi. (c) and (d) The SEM images corresponding to (a) and (b). The fresh thrombi tended to contain many randomly distributed RBCs and fibrin meshes. Aged thrombi only contain fibrin meshes and most RBCs are squeezed from thrombi.

**Figure 3:**
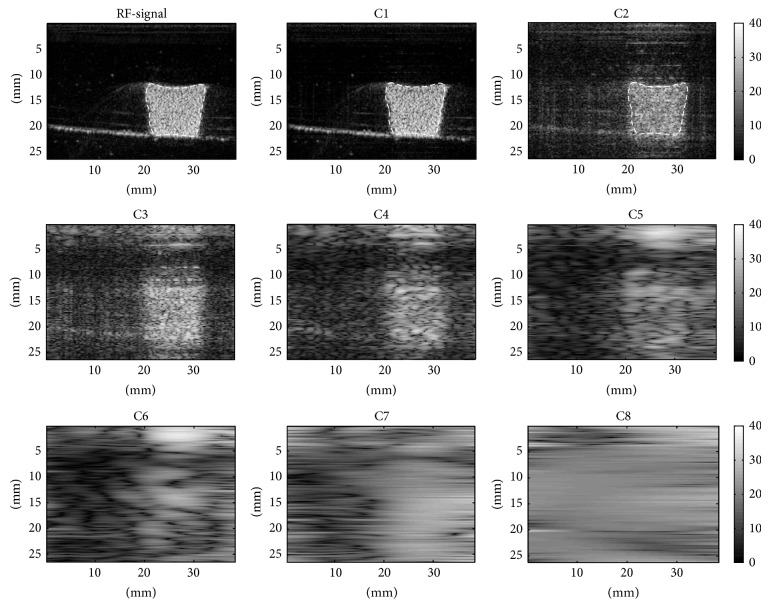
The B-mode images and the corresponding IMF images for fresh thrombi (described with white dashed lines). B-mode and IMF-based images are constructed using the log-compressed envelopes of the RF and IMF data obtained from the EMD.

**Figure 4:**
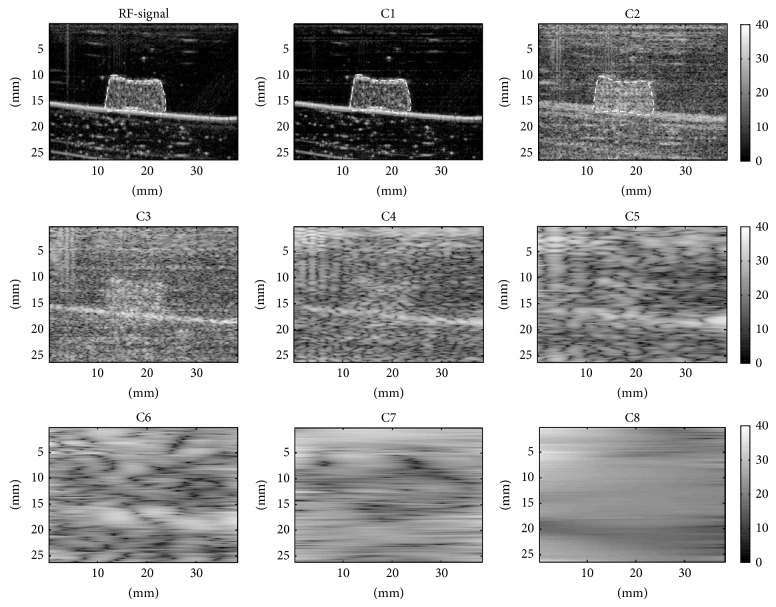
The B-mode images and the corresponding IMF images for aged thrombi (described with white dashed lines).

The representative H&E stain and SEM images of fresh and aged thrombi are presented in Figure 2. Fresh thrombi tend to contain many randomly distributed RBCs and fibrin meshes. For aged thrombi, few RBCs were observed in the thrombi. Both the H&E stain and SEM images were also examined by the pathologist to demonstrate that the number of RBCs decreased during aging and fibers gradually deposited in the thrombus. Figures 3 and 4 show the B-mode images and the corresponding IMF images for the fresh and aged thrombi (circled by white dashed lines), respectively. The speckle of the C1 image was similar to that of the B-mode image. This is because the first IMF was the datum with the highest-frequency components compared with the other IMFs. Therefore, the signal waveforms in the C1 image were similar to those of the original backscattered RF echoes but more symmetrical in the upper and lower envelopes [22]. This explains why the other IMF-based images (C3 and subsequent IMF images in Figures 3 and 4) exhibited poorer spatial resolutions.

**Figure 5:**
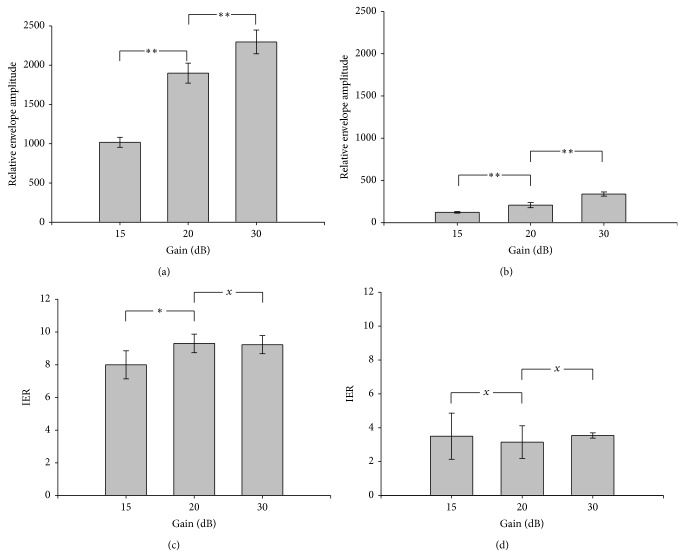
(a) and (b) The envelope amplitude of B-mode image obtained from different system gains for fresh and aged thrombi, respectively. (c) and (d) The IERs obtained from different gains for fresh and aged thrombi, respectively. The signal amplitude values of the B-mode and the IER decrease with increasing the thrombus age due to the decrease in the number of RBCs in the thrombus (^*^ *P* < 0.05; ^**^ *P* < 0.01; ^*x*^ *P* > 0.05).

Figures 5(a) and 5(b) show the envelope amplitude of B-mode image obtained from different system gains for fresh and aged thrombi, respectively. It was found that the envelope amplitude value of aged thrombus was smaller than that of fresh thrombus, representing that ultrasound B-scan has the ability to discriminate between fresh and aged thrombi. However, different system gains result in different measurement values (*P* value < 0.05). With increasing the system gain from 15 to 30 dB, the average envelope amplitude increased from 1000 to 2250 for fresh thrombi and from 120 to 350 for aged thrombi. Figures 5(c) and 5(d) show the IERs obtained from different gains for fresh and aged thrombi, respectively. Note that the IER also has the ability to differentiate fresh and aged thrombi. In particular, the IER is less affected by the system gain in detecting the thrombus age. In the range of system gain from 15 to 30 dB, the average IER varied between 8 and 10 for newly formed thrombi and around 3 for aged thrombi (*P* value > 0.05). The results demonstrated that the conventional B-scan can be used to describe thrombus aging by calculating the signal intensity. More importantly, the proposed IER is more independent of the system gain when using it to describe the change in the echogenicity of thrombus during aging.

## 5. Discussion

### 5.1. The Significance of This Study

Ultrasound grayscale image is always the first-line diagnostic tool for clinicians to examine the thrombus. This study demonstrated that using the signal intensity can discriminate between fresh and aged thrombi. The prerequisite for using the B-scan to identify thrombus aging is to remain the same system settings during measurements. However, this is not feasible clinically because different operators may have their preferred gains to adjust the appropriate image brightness they need. How to effectively reduce the gain effect on echogenicity measurement by B-scan is a fundamental but very critical issue. EMD, an adaptive signal decomposition method, has been widely applied in resolving several engineering and scientific problems [23]. Increasingly more studies have investigated EMD applications in the field of medical ultrasound imaging, including tissue harmonic imaging [24], signal filtering [25], improvements in tissue characterization by using statistical parameters [26, 27], image contrast enhancement [28, 29], and elastography construction [30]. In this paper, we utilized EMD to define the IER, which serves as a gain-independent index for discriminating between fresh and aged thrombi. The IER may allow an objective evaluation on the thrombus age using conventional B-mode image in clinical applications.

### 5.2. Effect of Thrombus Aging on Signal Intensity and the IER

In this study, the* in vitro* model was used to explore the relationship between the thrombus structure and the signal amplitude of ultrasound. As demonstrated by the results in Figure 2, histological observations on the structure of* in vitro* thrombi are consistent with those of* in vivo* thrombus models [14, 15], indicating that the number of RBCs in the thrombus decreases with aging. Thus, the thrombus that was induced by the proposed* in vitro* model could be used to simulate structural changes in the scatterer arrangement of the thrombus during aging. Ultrasound backscattered signals are typically formed from the summation of echo signals returned from each scatterer in a tissue. The number of RBCs determines the degree of wave interference and the corresponding signal intensity. This is the reason why the signal amplitude of B-scan for aged thrombi differs from that for fresh thrombi.

The current results demonstrated that the IER of the aged thrombus is smaller than that of newly formed thrombus. To explain why (*R*_C1_/*R*_C2_)_Aged_ < (*R*_C1_/*R*_C2_)_Fresh_, we tried to infer possible reasons from two facts as follows: (i) the signal amplitude of the C1 and C2 images decreases with increasing the thrombus age due to the decrease in the number of RBCs in the thrombus; (ii) according to the properties of EMD, the signal intensity of the C1 image is always larger than that of the C2 IMF. According to the above two points, one possible reason may be due to the fact that the signal intensity of the C1 image is more sensitive than that of the C2 image to the variation in the number of RBCs. The first C1 IMF adequately follows the form of the original ultrasound signal and is the highest-frequency component. It has been shown that the strength of ultrasonic scattering is proportional to the fourth power of frequency and sixth power of scatterer size [31]. Thus, during thrombus aging, high-frequency IMF component allows the detection of variations in the underlying physical processes with a higher sensitivity, resulting in a more significant decrease in the signal amplitude than low-frequency IMFs.

### 5.3. Comparison of the IER with Other Studies

The IER is an index obtained from signal self-decomposition and normalization. The concept of normalization was also applied in the previous studies. In the past, few studies have proposed that the harmonic-to-fundamental ratio (HFR) inhibits the confounding effects of attenuation in contrast echocardiography [32, 33]. During contrast echocardiography, ultrasound signals are backscattered from the microbubbles, thereby contributing to both fundamental and harmonic components. In HFR, the fundamental component is used to normalize the harmonic component in the frequency domain. A study reported that the spectral normalization in HFR can partially overcome the ultrasound signal attenuation in contrast echocardiography [33]. Compared with the HFR, the proposed IER is also an intensity ratio between two ultrasound signals with different frequency components (i.e., C1 and C2 modes obtained from EMD). However, the difference between the IER and the HFR-like parameters is that the signal normalization in IER is performed in the time domain. Furthermore, using additional contrast media to generate signals with different frequencies is unnecessary in the IER calculation because the EMD method adaptively decomposes image data into IMFs based on the characteristics of the signals.

### 5.4. Potential Applications and Future Work

Clinical thrombus treatments depend on the accurate determination of thrombus age. For instance, treatment planning for a fresh thrombus may require the injection of heparin despite substantial bleeding risk. In contrast, old thrombi may be treated with coumadin (oral anticoagulant) instead of heparin [4]. Invasive thrombus therapy may not yield favorable results if the thrombus is older than seven to ten days [3, 34]. Therefore, thrombus age determination may be useful in the evaluation of treatment efficacy [9]. The IER based on the conventional B-scan may be used to assist the establishment of personalized thrombolytic treatment planning when functional imaging systems are unavailable (e.g., elastography). Prior to clinical applications of the proposed method, few issues may require further investigation.

First, the dynamic range of the IER may be insufficient to reflect the slight changes in tissue echogenicity; thus, the sensitivity of the IER in detecting different echogenicities could be improved further. Second, the transducer determines the bandwidth of the received ultrasound signals. Therefore, different transducers may receive signals with varied bandwidths, thereby leading to different EMD outcomes (e.g., different frequency characteristics in each IMF) and indicating the importance of exploring the effects of transducer characteristics on the IER. Third, future studies could explore how to improve the computational efficiency of EMD. For example, parallel processing units (e.g., graphics processing unit, GPU) and programming language C may be used to accelerate the EMD computation [29].

## 6. Conclusion

This study proposed using EMD of ultrasound backscattered data as a strategy to identify the thrombus age by ultrasound B-scan image. The index IER defined as the signal intensity ratio calculated from the first and second IMF components obtained from EMD has been demonstrated to have the ability to discriminate between newly formed and aged thrombi. In particular, the IER measurement is less affected by the system gain. The experimental findings imply that the IER can provide clinicians with a relatively objective evaluation on the thrombus age. In the future, conventional ultrasound B-mode image integrated with the IER approach may be used to assist the establishment of personalized thrombolytic treatment planning.
